# Isolation and Identification of *Myo*-Inositol Crystals from Dragon Fruit (*Hylocereus polyrhizus*)

**DOI:** 10.3390/molecules17044583

**Published:** 2012-04-17

**Authors:** Ow Phui San Rebecca, Amru Nasrulhaq Boyce, Chandran Somasundram

**Affiliations:** Institute of Biological Sciences, Faculty of Science and Centre for Research in Biotechnology for Agriculture (CEBAR), University of Malaya, 50603 Kuala Lumpur, Malaysia; Email: amru@um.edu.my (A.N.B.); chandran@um.edu.my (C.S.)

**Keywords:** dragon fruit, crystals, *Hylocereus polyrhizus*, *myo*-inositol

## Abstract

Crystals isolated from *Hylocereus polyrhizus* were analyzed using four different approaches—X-ray Crystallography, High Performance Liquid Chromatography (HPLC), Liquid Chromatography Tandem Mass Spectrometry (LC-MS/MS) and Nuclear Magnetic Resonance (NMR) and identified as *myo*-inositol. The X-ray crystallography analysis showed that the unit-cell parameters were: *a =* 6.6226 (3) Å, *b =* 12.0462 (5) Å, *c =* 18.8942 (8) Å, *α =* 90.00, *β =* 93.98, *δ =* 90.00. The purity of the crystals were checked using HPLC, whereupon a clean single peak was obtained at 4.8 min with a peak area of 41232 μV*s. The LC-MS/MS technique, which is highly sensitive and selective, was used to provide a comparison of the isolated crystals with a *myo*-inositol standard where the results gave an identical match for both precursor and product ions. NMR was employed to confirm the molecular structure and conformation of the crystals, and the results were in agreement with the earlier results in this study. The discovery of *myo*-inositol crystals in substantial amount in *H. polyrhizus* has thus far not been reported and this is an important finding which will increase the marketability and importance of *H. polyrhizus* as a crop with a wide array of health properties.

## 1. Introduction

*H. polyrhizus* originates from Latin America [1] and is a member of the Cactaceae family which are mainly appreciated for their ornamental qualities. According to Le Bellec *et al.*, [2] there are at least 250 cultivated species of fruit-bearing and industrial crops in this drought resistant family. The exotic features of dragon fruit with its attractive deep purple coloured pulp make it highly appealing in the European and United States markets [3], and this crop is widely cultivated in Vietnam, Malaysia, Taiwan, China, Okinawa, Israel and Southern China. Studies have shown that a mature dragon fruit contains considerable amount of total soluble solids, and is rich in organic acids [4], protein [2], antioxidants [5] and other minerals like potassium, magnesium, calcium and vitamin C.

Inositol, a six carbon cyclitol, which is essential for the development of plants, animal and some organisms, comprises of stereoisomers designated as *myo*-inositol, cis-inositol, allo-inositol, epi-inositol, muco-inositol, neo-inositol and scyllo-inositol [6], where *myo*-inositol is the most widely distributed one in Nature. According to Loewus and Murthy [7], the first discovery of *myo*-inositol was in 1850 by Sherer from muscle extract and ever since then, many studies –have been carried out to establish the significance of this compound [8,9,10,11,12].

*Myo*-inositol, a six carbon cyclic polyalcohol is a precursor to biosynthesis of many compounds involved in phosphorus storage, signal transduction, stress, protection, hormonal homeostasis and cell wall biosynthesis in plants [13]. The significance of *myo*-inositol to animals was documented as early as 1979, when Whiting *et al.*, [8] discovered that a decrease in peripheral motor-nerve-conduction velocity is associated with a decrease in nerve *myo*-inositol content in diabetic rats. 

This discovery prompted many studies to establish the relationship between *myo*-inositol and its effects on human health. The functions and roles of *myo*-inositol in humans have been linked to bipolar disorder [14], production of L-chiro-inositol and D-chiro-inositol in insulin action [6], multiple sclerosis [15], Alzheimer’s disease [16] and regulation of the sorbitol pathway in diabetic patients [17]. The objective of this study was to identify the crystals isolated from the dragon fruit extract which have not been reported thus far.

## 2. Results and Discussion

### 2.1. Purification and Crystallization

**Figure 1:**
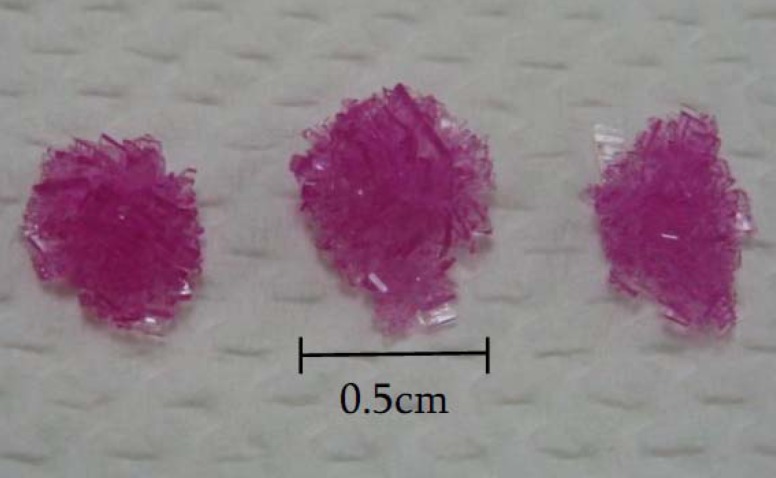
Example of crystals obtained after storage at 4–6 °C for 7 days.

**Figure 2:**
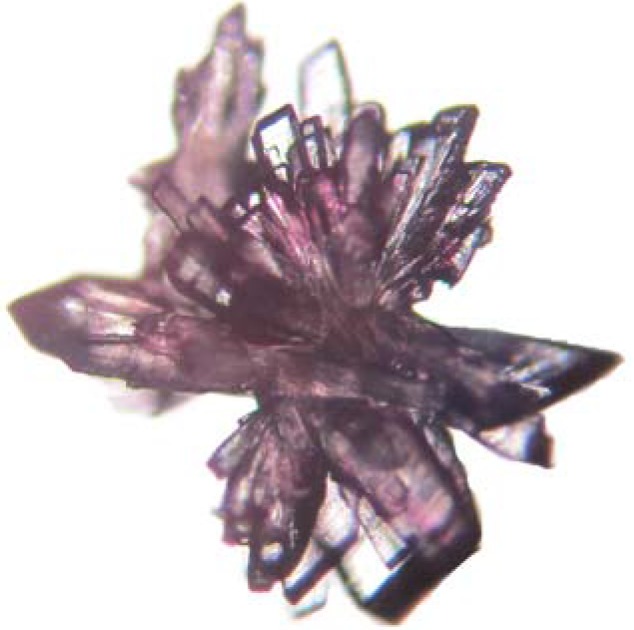
View of crystal under a microscope at 10×.

The yield of the crystals obtained after 7 days was about 2.0 g. Figure 1 shows an aerial view of the air-dried crystals before storage and Figure 2 show a 10× view under a microscope.

### 2.2. X-Ray Crystallograhy Analysis

**Figure 3:**
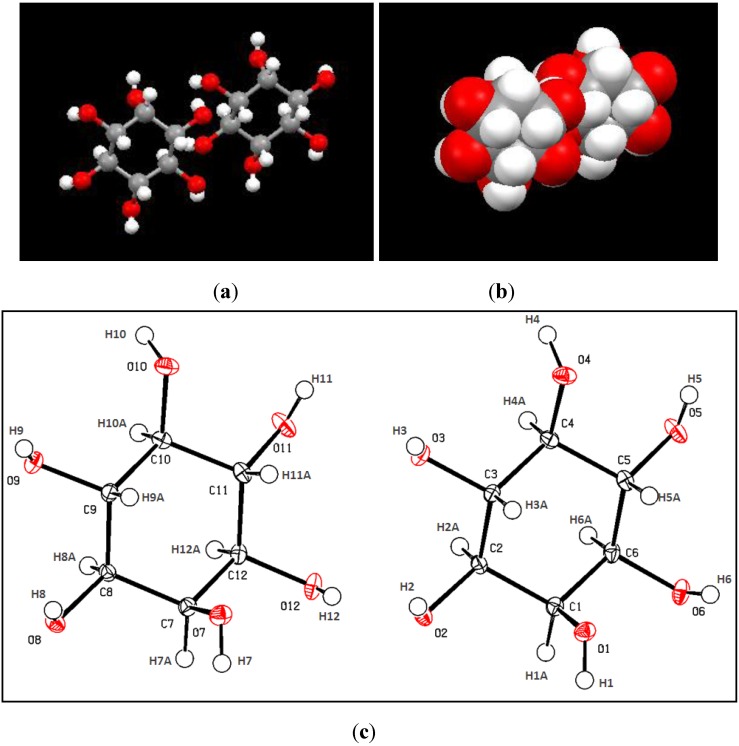
(**a**) Orthographic projection of the asymmetric unit of *myo*-inostiol along the *c* axis (**b**) A spacefilled orthographic projection of the asymmetric unit of *myo*-inostiol along the *c* axis (**c**) A complete labelled ellipsoid plot of the two units of the asymmetric unit *myo*-inositol (C_12_H_24_O_12_).

Unit-cell parameters, as measured with a Bruker SMART APEX diffractometer were: *a =* 6.6226 (3) Å, *b =* 12.0462 (5) Å, *c =* 18.8942 (8) Å, *α =* 90.00, *β =* 93.98, *δ =* 90.00. The crystal density was reported as 1.592 cm^−3^ while the crystal volume was recorded at 1503.69(11) Å^3^. These parameters are in good agreement with those reported by Rabinowitz and Kraut [18], indicating that the crystals isolated from dragon fruit pulp are *myo*-inositols. The chemical formula and molecular weight of the crystals were C_12_H_24_O_12_ and 360.31, showing that there are two units of *myo*-inositol per asymmetrical unit. This is further supported by the plots in Figure 3 which were generated using the enCIFer Software from CCDC. From the X-Ray analysis, the crystals were reported have monoclinic cell setting with P2_1_/n space group while the unit cell dimension is 0.35 × 0.25 × 0.15. All the cell parameters results were obtained from 18816 reflections with wavelength set at Mo *K*α radiation, λ = 0.71073 Å using a Graphite monochromameter. As X-ray crystallography is able to determine the arrangement of atoms and chemical bonds within a crystal producing a three dimensional representation based on electron density, it remains the chief method for characterizing atomic structure of many compounds and resolving new materials.

Looking at the result, which is in good agreement with earlier X-ray crystallography reports of the unique parameters and chemical characteristics of *myo*-inositol, the subsequent analytical methods are essential to ensure the purity of the crystals obtained and to confirm a definite identification.

### 2.3. Qualification of Crystal Purity Using High Performance Liquid Chromatography

**Figure 4:**
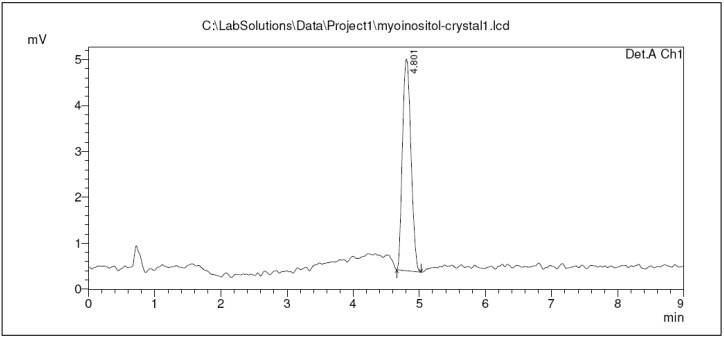
Crystal purity check using HPLC where the peak and retention time of the *myo*-inositol sample was observed at 4.8 min.

In the crystal purity qualification using HPLC (Figure 4), only one peak was detected in the chromatogram, indicating that the isolated *myo*-inositol crystals do not contain any other compounds or suffers any contamination during the experiment. 

The sample peak was observed at 4.8 min with a peak area of 41232 μV*s. The result from this purity check was a qualification to proceed later on to using Liquid Chromatography Tandem Mass Spectrometry (LC-MS/MS) for a more accurate analysis on the crystals. Since HPLC is more sensitive compared to other chromatographic methods as it can detect low concentrations of compounds, as low as nanogrammes [19], this method was employed to confirm the purity of the crystals from *H. polyrhizus* before putting the sample through LC-MS/MS with a highly sensitive system to avoid possible column contamination or confusion in identifying the sample. 

### 2.4. Liquid Chromatography Tandem Mass Spectrometry (LC-MS/MS) Analysis

**Figure 5:**
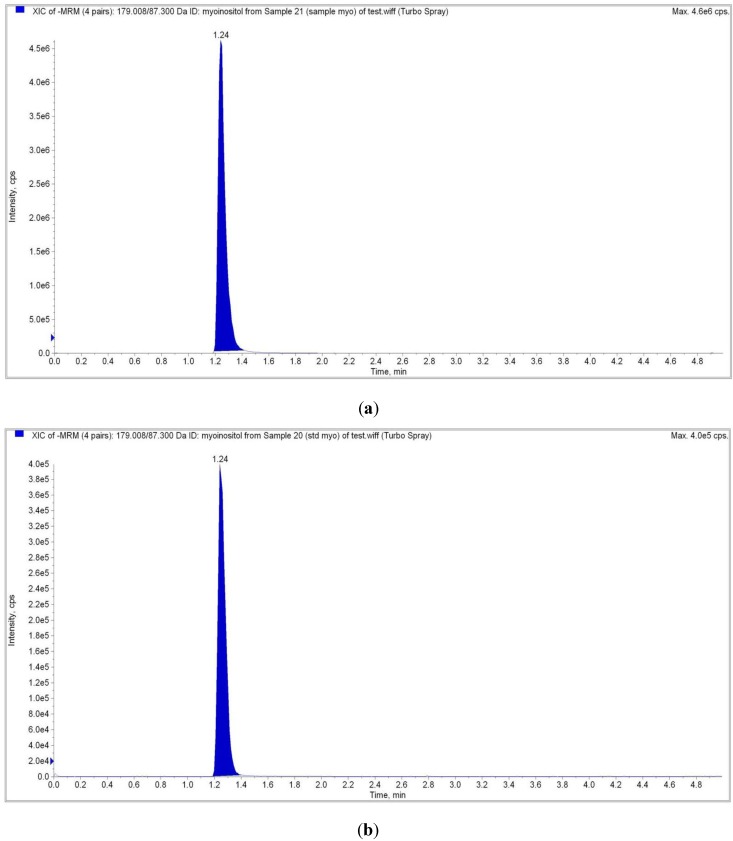
(**a**) LC-MS/MS analysis on crystals isolated from red dragon fruit where the peak and retention time was observed at 1.2 min with a 179*m/z* precursor ion; (**b**) LC-MS/MS analysis on *myo*-inostiol standard where the peak and retention time was observed at 1.2 min with a 179 *m/z* precursor ion which is identical to the crystal sample from red dragon fruit.

In the LC-MS/MS analysis, a mass spectrum was first established using a *myo*-inositol standard and the known mass-to-charge (*m/z*) ratio of *myo*-inositol was used as a benchmark throughout the experiment using the positive ion mode [20,21]. The precursor ion peak from the *myo*-inositol standard mass spectrum was at 179 *m/z* and its product ion at 87 *m/z* (Table 1) In the crystal sample, the precursor ion peak was observed at 1.24 min with a mass spectrum of 179 *m/z* [Figure 5(a)] which was obtained alongside its product ion, 89 *m/z* (Table 1).

**Table 1:** Summary of the LC-MS/MS analysis on the crystals from red dragon fruit and *myo*-inostiol standard.

Analyte Peak Name	Analyte RT	Precursor ion (*M/Z*)	Product Ion (*M/Z*)
Sample	1.24	179.00	87.00
*Myo*-inositol Standard	1.24	179.00	87.00

This result was an exact match to the peak produced by the *myo*-inositol standard [Figure 5(b)]. The Liquid Chromatography Tandem Mass Spectrometry (LC-MS/MS) technique, which is sensitive, specific and accurate [22] was employed to establish a reliable mass spectrum. First a *myo*-inositol standard was used to establish the reference mass spectrum and to obtain the distinctive precursor and product ion for this compound. As observed in Figure 5, the crystal sample showed an identical match to the *myo*-inositol standard. This leads to the final qualification technique, the Nuclear Magnetic Resonance (NMR) analysis. 

### 2.5. NMR Analysis

**Figure 6:**
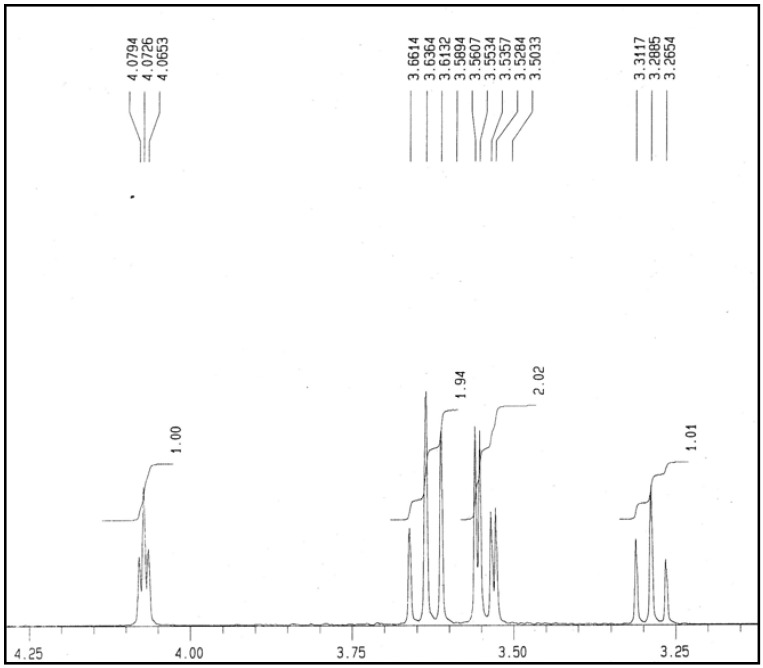
^1^H-NMR spectrum obtained from analysis on *myo*-inositol crystals isolated from red dragon fruit.

**Figure 7:**
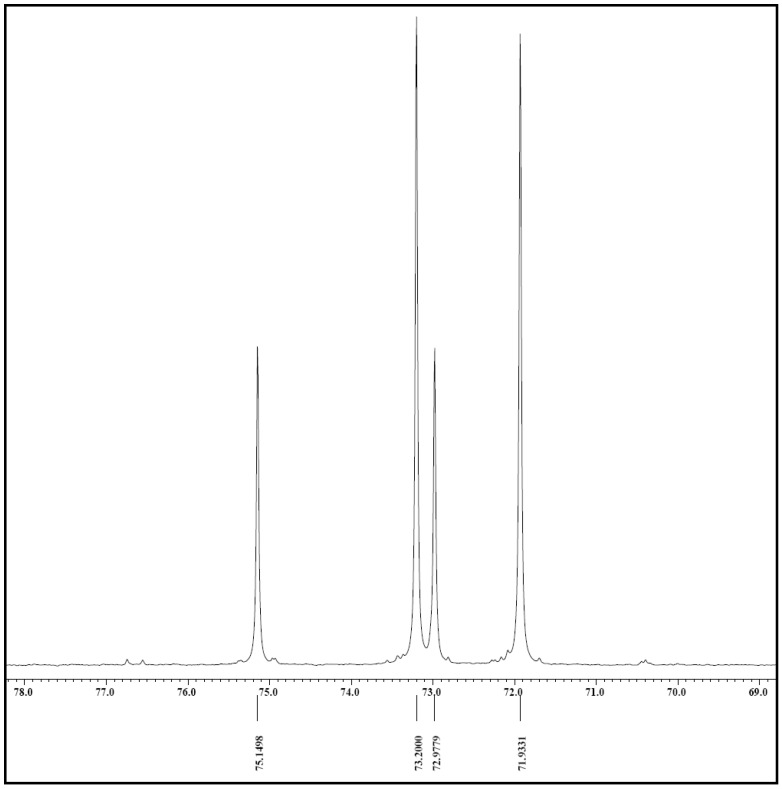
^13^C-NMR spectrum obtained from analysis on *myo*-inositol crystals isolated from red dragon fruit.

In this study, the one dimensional ^1^H-NMR was used to confirm the purity of the *myo*-inositol crystals and to observe the chemical shifts of the six ring proton resonance. The ^1^H-NMR spectrum shown in Figure 6 is again in agreement with the structure of one unit of *myo*-inositol (C_6_O_12_H_6_) with the relative intensities of 1:2:2:1. The probable assignment for the group of signals observed in the spectrum based on their chemical shifts and coupling constants are as follows: H2–H4–H6–H1–H3–H5 which is closely similar as reported by Cerdan *et al.*, [23] and Barrientos and Murthy [9]. The ^13^C-NMR spectrum of the crystal sample (Figure 7) gave rise to four prominent signals in a clean zone of 75.15, 73.20, 72.98 and 71.93 ppm with relative intensities of 1:2:1:2 which probably corresponds to carbons C5–C1–C3–C2–C4–C6 [23]. An important aspect derived from the ^1^H- and ^13^C-NMR analysis in this study is the purity and qualitative information about the molecular formula of the *myo*-inositol crystals from the pulp of *H. polyrhizus*. In terms of quantitative and exact assignment of each signal on the spectrums, validation by comparison with other techniques in needed but in this study, the purpose is only to identify the crystals. 

Accordong to a review by Loewus and Murthy [7], *myo*-inositol is highly involved in plant metabolism and the following list is just to name a few processes in which it is involved: seed dessication, osmo-regulation, nutrient storage, membrane biogenesis, senescence, auxin physiology, fertilization as well as synthesis of cell wall uronosyl and pentose units. While this list make *myo*-inositol a crucial compound in regulating plant biochemistry and physiology, more importantly, it has also been found concentrated in cerebrospinal fluid in the range of 100–500 μM and it increases to 10 mM or more in brain cells [24]. The probable reason given to this phenomenon is that *myo*-inositol actively participates in the synthesis of membrane phospholipids which affects neuronal plasticity and synapse formation in neuron cell [14]. This is where Shimon *et al.*, [25], and Silverstone *et al.*, [10] reported the possible connection between inositol depletion in the frontal temporal lobes and patients with bipolar disorder, where a depletion in inositol would result in mitochondria dysfunction which then causes a decrease in oxidative phosphorylation, a decrease in intracellular pH and an increased level of lactate in the brains causing neural related disorders. Other roles of inositol and its common derivative *myo*-inositol, include as potent regulators for a large number of hormones, growth factors and neurotransmitters. As a result its significance, *myo*-inositol has been used as part of treatment for diabetes mellitus [26]; obsessive-compulsive disorder [27]; status epilepticus [11]; psoriasis and eczema; and recently a report that *myo*-inositol may improve metabolic syndromes in postmenopausal women [12].

*Myo*-inositol was reported to be commonly found in citrus fruits, beans, grains and nuts as early as 1980 by Clements and Darnell [28]. In their study on 487 different types of food, the highest amount of *myo*-inositol were found in 4.40 mg/g of *myo*-inositol in *Phaseolus vulgaris*, 4.07 mg/g in *Prunus domestica*, 3.55 mg/g in *Cucumis melo*, 3.07 mg/g in *Citrus sinensis*, 2.83 mg/g in *Pisum sativum*, 2.78 mg/g in *Prunus dulcis*, 2.74 mg/g in bran, 1.34 mg/g in *Arachis hypogaea and* 0.42 mg/g in oatmeal. In this study, 2 g of *myo*-inositol was recovered from 450 g of *H. polyrhizus* pulp and this translates to about 4 mg/g of *myo*-inositol in one fruit, which is almost similar to its content in *Phaseolus vulgaris*, a very important global agricultural produce. As mentioned earlier, *myo*-inositol has been reported to be involved in osmo-regulation in many plants including those as previously reported by Ishitani *et al.*, [29] in *Mesembryanthemum crystallinum* (ice plant); and RayChaudhuri and Majumber [30] in *Oryza sativa L.* (rice). Since *H. polyrhizus* is a cactus which thrives in an arid environment, osmo-regulation which controls a plants’ reaction to environmental stresses including drought, salinity and extreme temperature [31] is even more crucial and thus, this amount of *myo*-inositol found in *H. polyrhizus* is not surprising at all. This finding of *myo*-inostiol, an essential compound for many metabolic processes in organisms puts *H. polyrhizus* in a position as a new alternative for many health/diet recommendations and as a potential source for pharmaceutical products.

## 3. Experimental

### 3.1. Sample Preparation

Fruits were purchased from local market situated 5 km from the laboratory. Fruits were halved and peeled manually. The sample preparation, purification and crystallization methods were adopted according to Saito *et al.*, [32] with modifications of sample weight and chemical volumes in this study due to the difference in sample and starting material. 

Fruit pulp (450 g) was immersed in 60% methanol (500 mL) and left to stand at room temperature (24 ± 2 °C) for 2–3 h. Sample was warmed at 60–70 °C in a water bath for a final 20 min and the dragon fruit pulp was removed by filtering sample through one layer of mira cloth. The filtrate was collected and used for subsequent experiments. 

### 3.2. Purification

The filtrate (500 mL) was concentrated *in vacuo* at 40–50 °C using a rotary evaporator (Büchi R-210) for 150 min into a slurry syrup. The slurry was thoroughly washed with ether (100 mL) and then 100% ethanol (100 mL). The solvent was decanted carefully and the insoluble portion was dissolved in water (30 mL). The sample was filtered using mira cloth and 100% ethanol (30 mL) was added to the filtrate. Sample was kept at 4–6 °C for 3–4 h and filtered using mira cloth to remove any impurities. The filtrate obtained was evaporated to dryness *in vacuo* at 40–50 °C. 

### 3.3. Crystallization

The resulting mass was dissolved in cold water (25 mL) and 100% ethanol (50 mL). The sample was left at 4–6 °C for 7 days for crystallization. The resulting crystals were removed from solution by filtering through one layer of mira cloth, air dried and stored in a glass vial. 

### 3.4. Crystal structure Determination Using X-Ray Crystallography

Data for the crystal structure was collected on a Bruker SMART APEX diffractometer by using a SADABS-Bruker Nonius detector. For all measurements, graphite-monochromated Mo Kα radiation was used. The structure was solved by direct methods [33] and refined [34] by full-matrix least squares on *F*^2^ (all data) by using the SHELXL-97 program package. All non-hydrogen atoms were refined anisotropically. The H atoms bound to C aromatic atoms were refined using a riding model with C–H = 0.93 Å U_iso_(H) = 1.2 U_eq_(C). The remaining H atoms were located from different Fourier map and refined isotropically with U_iso_(H) = 1.2 U_eq_ (C or O). Crystallographic data (excluding structure factors) for the structure in this paper have been deposited with the Cambridge Crystallographic Data Centre (CCDC) and was allocated the deposition number CCDC-784857.

### 3.5. Qualification of Crystal Purity Using High Performance Liquid Chromatography

HPLC was carried out by using an LC-10A UFLC system with a SIL-HT automatic sample injector (Shimadzu, Kyoto Japan) with a UV/VIS detector (SPD-20A) equipped with a 2.1 × 150 mm Zorbax Eclipse XDB C18 3.5 µm column. Mobile Phase A was deionised water and mobile phase B was 0.1% formic acid in methanol running with gradient mode. The gradient program began with 95% B, ramped to 5% B at 3 min and then return to 95% B at 3.01 min and this condition was held for further 9.00 min.The detection was set at 546 nm and 10 μL of sample was allowed to elute through the system for 9 min at a flow rate of 0.3 mL/min and column temperature was set to 40 °C.

### 3.6. Liquid Chromatography Tandem Mass Spectrometry (LC-MS/MS) Analysis

The LC-MS/MS system consisted of an LC-10A UFLC system with a SIL-HT automatic sample injector (Shimadzu, Kyoto Japan) and an API 5500 Q-Trap LC-MS/MS system (Applied Biosystems, Lincoln Centre Drive, Foster City, CA, USA). The separations were performed on a 2.1 × 150 mm Zorbax Eclipse XDB C18 3.5 µm column. A gradient elution was employed on the column at 0.25 mL/min with mobile phase A (deionised water) and mobile phase B (0.1% formic acid + HPLC grade methanol) running a linear gradient in which the percent of mobile phase B was held at 10% for 0.10 min, then linearly increased to 70% during the next 1.90 min. The mobile phase B was held at 90% for 0.50 min and then immediately returned to 10%. The analysis time was 5.0 min per sample. The typical injection volume was 1.0 µL. All the mass spectra were collected on a linear ion trap quadrupole LC/MS/MS mass spectrometer. The Turbo Ion Spray (TIS) interface was operated in positive ion mode and temperature was maintained at 350 °C. Unit mass resolution was used in all the experiments. LC-MS/MS data were acquired using Analyst Software Version 1.5.1.

### 3.7. Nuclear Magnetic Resonance (NMR) Analysis

The obtained crystal was dissolved in 0.75 mL of D_2_O (99.996% ^2^H; Merck) and the solutions were filled into 5 mm-diameter NMR tubes. All spectra were accumulated at room temperature (24 ± 2 °C) using a JEOL LAMBDA 400 NMR spectrometer equipped with a Delta software. The resonance was 399.8 MHz for ^1^H and 100.5 MHz for ^13^C.

## 4. Conclusions

The experiments carried out showed that the crystals which were obtained in abundance from the pulp of *H. polyrhizus* are *myo*-inositol, which is a very important and significant chemical component to human health, plant metabolism and many industries especially the pharmaceutical industry and medical field. This discovery and isolation of *myo*-inositol from this crop further raise the status of *H. polyrhizus* in the market from just being an exotic fruit crop and on top of the existing nutritional properties; *H. polyrhizus* now has an extra value added quality to be a good source as health food. Further analysis and work on this report and this crop should be continued as *H. polyrhizus* is a valuable crop with significant amount of health properties which is beneficial for consumers.
